# Unmet healthcare needs among the population aged 50+ and their association with health outcomes during the COVID-19 pandemic

**DOI:** 10.1007/s10433-023-00758-x

**Published:** 2023-04-29

**Authors:** Carlota Quintal, Luis Moura Ramos, Micaela Antunes, Óscar Lourenço

**Affiliations:** CeBER, Faculty of Economics, Univ Coimbra, Avenida Dias da Silva, 165, 3004-512 Coimbra, Portugal

**Keywords:** Unmet needs, Mortality, Cancer, Self-assessed health, COVID-19, SHARE

## Abstract

The COVID-19 pandemic led to unprecedented levels of subjective unmet healthcare needs (SUN). This study investigates the association between SUN in 2020 and three health outcomes in 2021—mortality, cancer, and self-assessed health (SAH), among adults aged 50 years and older, using data from the regular administration of the Survey of Health, Ageing and Retirement in Europe and from the two special waves administered in 2020 and 2021 regarding COVID-19. Three types of SUN were surveyed: care foregone due to fear of contracting COVID-19, pre-scheduled care postponed, and inability to get medical appointments or treatments demanded. We resort on the relative risk and the logistic specification to investigate the association between SUN and health outcomes. To avoid simultaneity, 1-year lagged SUN variables are used. We found a negative association between SUN and mortality. This result differs from the (scarce) previous evidence, suggesting that health systems prioritised life-threatening conditions, in the pandemic context. In line with previous studies, we obtained a positive association between SUN and worse health, in the case of cancer, though it is statistically significant only for the global measure of SUN (any reason). The higher chances of reporting cancer among those exposed to SUN might mean delayed cancer diagnosis, confirming that healthcare foregone was truly needed for a timely diagnosis. The association between SUN and poor or fair SAH is positive but not statistically significant, for the period analysed.

## Introduction

Self-reported unmet healthcare needs, also known as subjective unmet needs (SUN) (Allin et al. 2010), have become an essential indicator of access to healthcare (Allin and Masseria 2009; EXPH 2017; Thomson et al. 2019). Factors associated with SUN are widely explored by now, namely in European countries. Individuals with low income, worse health, of younger ages, immigrants, unemployed, and women tend to present higher levels of SUN for general healthcare (Baert and De Norre 2009; Israel 2016; Röttger et al. 2016; Fjaer et al. 2017). Lack of private health insurance might lead to an increased risk of SUN (Connolly and Wren 2017), while it seems irrelevant whether persons are registered at public or private primary care providers (Lindström et al. 2018). Differently, higher levels of trust (Lindstrӧm et al. 2017) and social capital in general (Quintal et al. 2019) are negatively associated with SUN. At the country level, unmet needs for medical care seem to be higher in countries with larger income inequalities (Israel 2016) as well as in countries where out-of-pocket payments weigh more on total health expenditure (Chaupain-Guillot and Guillot 2015).

Although access to healthcare merits investigation on its own, a strong concern with unmet needs stems from the possibility of unmet healthcare needs leading to a deterioration of the individuals’ health (Aragon et al. 2017). Contrasting with the numerous studies on predictors of SUN, empirical analyses of the health implications of SUN are much scarcer particularly in Europe, but also elsewhere. Existing evidence suggests that SUN in the past cause a worsening in self-assessed health (SAH) in the present. One study obtained this evidence for Canada (Gibson et al. 2019) and two others for Korea (Ko 2016; Kim et al. 2019), considering one-year or two-year lagged SAH. Some studies used mortality, instead, as the health outcome variable, concluding that unmet needs were associated with increased mortality in a five-year follow-up period for the case of Sweden (Lindström et al. 2020) and a three-year follow-up period for the case of Chinese elderly (Zhen et al. 2015). However, in the latter study, unlike in the previous ones, unmet needs concerned assistance in performing activities of daily living and not specifically healthcare. An early study by Alonso et al. (1997) also found that unmet healthcare needs were associated with an increased risk of mortality for the elderly (Spain). Nonetheless, in this study, direct questions about unmet needs were not available. Hence, the authors considered that individuals with bad health, who reported no visits to/from a physician in the previous twelve months, had unmet healthcare needs. Besides SAH and mortality, an association between SUN in one period and worse health, measured by chronic conditions as well as activity limitations, in the following period, was obtained by Gibson et al. (2019). Using a broader health outcome variable, some studies produced evidence of a negative relationship between SUN and health-related quality of life (Ko 2016; Ju et al. 2017; Gibson et al. 2019). Finally, a few studies focused on specific clinical outcomes mostly related with mental health, such as Lasalvia et al. (2005), Gaugler et al. (2005), and more recently Stein et al. (2019). These studies too found a negative association between unmet needs and good health, but needs were assessed in a different way, adapting the Camberwell Assessment of Need, consisting of health and social needs across various domains.

Previous evidence further suggests that the magnitude of the association between SUN and health-related quality of life is more pronounced in economically vulnerable groups (Ju et al. 2017). There is also evidence that the magnitude of the association between SUN and SAH is bigger for unmet needs due to economic reasons rather than any other reasons, and smaller in the case of mild symptoms (Ko 2016). In light of these results, it is possible that more educated individuals are more capable of identifying milder conditions for which healthcare can be foregone without serious health consequences. On the other hand, poorer individuals might not have the means to meet healthcare needs even when they recognise them as serious needs.

All the above studies used data collected before the COVID-19 pandemic. The pandemic created a totally different panorama, with a massive reduction in healthcare utilisation (Moynihan et al. 2021). Before the pandemic, surveys and analyses of unmet healthcare needs were mainly concerned with barriers to access to healthcare, particularly related to costs and waiting times. With the outbreak of COVID-19, health systems reallocated healthcare resources to COVID-19 patients, leading to the cancellation and/or postponement of many planned treatments. Patients themselves, either due to fear of infection or to avoid burdening health services, have restrained demand. Measures as lockdowns and stay-at-home orders have also affected healthcare utilisation (Moynihan et al. 2021). Using data from a special wave of the Survey of Health, Ageing and Retirement in Europe (SHARE), the SHARE Corona Survey launched in 2020 (SC1), some studies have addressed unmet healthcare needs in the first months of the pandemic. Some of the usual predictors of SUN were found in these studies such as being female, having poor health and a bad economic situation (Smolić et al. 2021). Another study reports that the impact of economic vulnerability was stronger among those who were in poor health before the outbreak (Arnault et al. 2021). At the country level, postponed medical care is associated with more stringent governmental anti-COVID-19 measures (Jiskrova et al. 2021). Comparisons with previous analyses of unmet needs must be however cautious because some of the unmet needs, in SC1, were assessed by asking individuals if their appointments were cancelled/postponed. Thus, in this case, unmet needs can only be observed among those individuals with scheduled appointments. However, irrespective of which factors are associated with unmet healthcare needs and of any limitation that might apply, one important question remains: what are the health implications of these unmet needs which emerged during the pandemic?

Bergeot and Jusot (2022) investigated the effect of unmet needs during the first wave of the pandemic on health outcomes (fear of falling, falling, fatigue, dizziness) up to one year after, relying on data from the two special waves of the SHARE Corona surveys collected in 2020 and 2021 (SC1 and SC2, respectively). These authors found a positive association between SUN in 2020 and all outcomes in 2021.

In the current study, we too take advantage of the data collected in the special waves of the SHARE Corona Survey, SC1 and SC2, to assess the association of unmet healthcare needs reported in 2020 with health outcomes reported in 2021, among the general population, aged 50 years or above. As in most studies analysing the association between SUN and health, two main health outcomes here analysed are SAH and mortality. In addition, we also analyse the association between SUN in 2020 and cancer incidence in 2021. Our health outcomes thus differ from those analysed by Bergeot and Jusot (2022).

A usual difficulty in linking SUN with health outcomes lies in the subjectivity of this measure. Individuals who value health more or who have higher expectations towards health services might be more prone to report unmet needs (Ko 2016). Hence, SUN might not represent true unmet needs given that they are based on respondents’ perceptions of need and are not based on evidence regarding the effectiveness of healthcare foregone (Gibson et al. 2019). In the context of the pandemic, because we are dealing with the cancellation/postponement of, or non-attendance to, planned appointments, one might say that most self-reported unmet healthcare needs are clinician-validated (Allin et al. 2010). Nonetheless, overuse and waste in the healthcare sector is well documented (Mafi and Parchman 2018), meaning that even clinician-validated SUN might not represent true unmet needs. There are already claims that not all healthcare foregone during the pandemic was necessary and that the pandemic context was an opportunity to reduce waste (Moynihan et al. 2020; Sorenson et al. 2020). There are mixed signals about the impact of the pandemic on the health of patients suffering from conditions not related with COVID-19. On the one hand, there is evidence of excess population mortality, in addition to deaths from COVID-19 (Lai et al. 2020) and increases in out-of-hospital cardiac arrests and contacts with emergency phone lines (Marijon et al. 2020; Perlini et al. 2020). On the other, it seems that cuts in healthcare utilisation were stronger for less severe forms of illness (Moynihan et al. 2021). The research about the association between unmet needs, in 2020, and health outcomes such as SAH and mortality, in 2021, might shed some light on this issue.

With the present study we aim to contribute to the very scarce literature on the association between SUN and health, especially in European countries. In addition, as frequent users of healthcare, older people were at an increased risk of unmet needs during the pandemic. Hence, we also aim to contribute to a more comprehensive understanding of the consequences of the pandemic, due to healthcare foregone, for the health of older patients suffering from conditions not necessarily related with COVID-19.

## Materials and methods

### Data

Data for this study come from the SHARE project. SHARE is a longitudinal study that includes eight ordinary waves of biennial surveys starting at 2004 and finishing at 2018/2019 (Börsch-Supan et al. 2013). The target population of SHARE consists of all persons aged 50 years or older who at the time of the interview had their domicile in a country that was part of the SHARE project (28 countries, including all European Union countries, except Ireland, plus Switzerland and Israel). The multidisciplinary, cross-national and longitudinal database contains individual-level data on health, demographic and socio-economic status, household structure, and social networks, for more than 123,000 individuals (*SHARE webpage*). Furthermore, it includes two special waves, one from 2020 and another from 2021, designed and applied to collect data about the social, health and economic impact of the COVID-19 pandemic (Scherpenzeel et al. 2020). Notwithstanding the differences among countries, the most frequently used sampling methodology is stratified multi-step random sampling. Although the individual participation rate varies from country to country, the overall participation rate is found to be systematically above 45% for every wave (Bergmann et al. 2019).

The special data sets collected in 2020 (SC1) and 2021 (SC2) (Börsch-Supan 2022a, b), covering the pandemic period, are the main data contributors for this analysis (the information about the specificities of SC1 and SC2 can be found in the SHARE Corona Release Guide, available at https://share-eric.eu/fileadmin/user_upload/Release_Guides/SHARE_Corona_Survey_Release_Guide.pdf). Both surveys were designed to collect data reflecting the living context brought about by the COVID-19 crisis. In both surveys, data were collected via computer-assisted telephone interview between June and August 2020 (SC1) and, one year later, between June and August 2021 (SC2). Despite the change from face-to-face-interviewing to telephone interviews, this was done in a way to minimise the drop of the retention rates between waves. Most countries hence achieved or even surpassed their retention rates from before the pandemic (Bergmann et al. 2022). The topics covered by SC1 and SC2 were essentially the same as those of the regular SHARE questionnaire (health and health behaviour; mental health; infections and healthcare; changes in work and in the economic situation; and social networks), but on a shortened version and more oriented to the living situation during the pandemic. SC1 interviewed 57,559 individuals while SC2 interviewed 49,253. However, for our analysis, we are interested in those subjects with valid cases in the two waves, and in those who were interviewed in SC1 and died before the second interview. Our final working database has 48,356 individuals with valid interviews in SC1 and SC2, plus 1199 individuals who were interviewed in 2020 and died before the 2021 interview. These are the sample sizes of the original datasets supplied by SHARE, however, due to the existence of missing values for some of the variables of interest, or to the need to select some sub-populations, the statistical analysis is based on a lower number of observations. Throughout the paper, we will provide the number of cases on which each analysis is based.

### Variables

There are three sets of variables—health outcome measures, SUN as measures of exposure, and control variables. Table 1 presents the definition of each variable used in the analysis and the Appendix provides additional information on which waves of SHARE were used, and how, to construct each variable. We analyse the effect of SUN on three health outcomes—cancer, mortality, and poor or fair SAH. Regarding SUN, there are three categories of unmet healthcare needs as shown in Table 1. Healthcare foregone due to fear of infection, SUN (Fear), which includes planned care but also applies to other situations. In the case of planned care, one might say that it represents the concept of clinician-validated SUN (Allin et al. 2010), corresponding to an objective unmet healthcare need. For other situations, SUN (Fear) can be regarded as ‘subjective, chosen unmet need’ (Allin et al. 2010). SUN (Postponement) fits the previous description of planned care. In the case of SUN (Unavailable care), individuals explicitly sought healthcare, which depends on their perceptions of need and expectations regarding health services. We also consider a global measure of unmet healthcare needs, irrespective of the reasons—SUN (Global). The control variables are defined in Table 1 (see also the Appendix for more details).

**Table 1:** Definition of variables

Variable	Definition
*Health outcome measures* (measured in SC2)	
Cancer	= 1 if individual selects, in 2021, from a list of health conditions, the option: “Cancer or malignant tumor, including leukemia or lymphoma, but excluding minor skin cancers”; 0 otherwise
Mortality	= 1 if individual was inquired in the SC1 wave and died afterwards, before SC2 interview; 0 otherwise
SAH Poor|Fair_Pand	= 1 if individual reports self-assessed health, in 2021, as poor or fair; 0 otherwise
*SUN as measure of exposure* (measured in SC1, at baseline)	
SUN (Fear)	= 1 if individual answers ‘yes’ to the question “Since the outbreak of Corona, did you forgo medical treatment because you were afraid to become infected by the coronavirus?”; 0 otherwise
SUN (Postponement)	= 1 if individual answers ‘yes’ to the question “Did you have a medical appointment scheduled, which the doctor or medical facility decided to postpone due to Corona?”; 0 otherwise
SUN (Unavailable care)	= 1 if individual answers ‘yes’ to the question “Did you ask for an appointment for a medical treatment since the outbreak of Corona and did not get one?”; 0 otherwise
SUN (Global)	= 1 if individual answers ‘yes’ to any of the questions identified in SUN (Fear), SUN (Postponement) or SUN (Unavailable care); 0 otherwise
*Control variables* (measured in SC1)	
Male	= 1 if male; 0 otherwise
Age	Age in years
Educ_low	= 1 if individual completed at most basic education (ISCED 1997 codes 0, 1, or 2); 0 otherwise
Educ_med	= 1 individual completed secondary or post-secondary education (ISCED 1997 codes 3 or 4); 0 otherwise
Educ_high	= 1 if individual completed tertiary education (ISCED 1997 codes 5 or 6); 0 otherwise
Income	Monthly equivalent income, before Corona outbreak, in thousand euros
Lives alone	= 1 if individual belongs to a one-person household; 0 otherwise
Big city	= 1 if individual lives in a big city; 0 otherwise
Suburbs	= 1 if individual lives in the suburbs or outskirts of a big city; 0 otherwise
Large town	= 1 if individual lives in a large town; 0 otherwise
Small town	= 1 if individual lives in a small town; 0 otherwise
Rural	= 1 if individual lives in a rural area or village; 0 otherwise
SAH Poor_Pre_Pand^a^	= 1 if individual reports self-assessed health as poor; 0 otherwise
SAH Fair_Pre_Pand^a^	= 1 if individual reports self-assessed health as fair; 0 otherwise
SAH Good_Pre_Pand^a^	= 1 if individual reports self-assessed health as good; 0 otherwise
SAH Very Good_Pre_Pand^a^	= 1 if individual reports self-assessed health as very good; 0 otherwise
SAH Excellent_Pre_Pand^a^	= 1 if individual reports self-assessed health as excellent; 0 otherwise
n_Chronic	Number of chronic conditions (from a list of 17 conditions)
Diabetes	= 1 if individual has diabetes; 0 otherwise
Country C^b^	= 1 if individual lives in country C; 0 otherwise

*SC1* SHARE Corona Survey 2020, *SC2* SHARE Corona Survey 2021

^a^In SC1, individuals were asked about their health before the Corona outbreak

^b^*List of countries* Austria, Belgium, Bulgaria, Croatia, Cyprus, Czechia, Denmark, Estonia, Finland, France, Germany, Greece, Hungary, Israel, Italy, Latvia, Lithuania, Luxembourg, Malta, Netherlands, Poland, Portugal, Romania, Slovakia, Slovenia, Spain, Sweden and Switzerland

### Statistical strategy

The main challenges to discerning the effect of SUN on individuals’ health status are dealing with unobserved heterogeneity and simultaneity between health status and SUN (Ko 2016; Gibson et al. 2019). One possible strategy to deal with the simultaneity issue is to conceptualize the special waves of SHARE as a prospective cohort study design. The cohort design allows the study of the individuals who have been exposed to SUN, followed over one year, with cancer, poor/fair SAH and mortality as health outcome measures, being compared, at the end of the period, with the group formed by those who have not been exposed to SUN. The 2020 wave (SC1) is the baseline and SC2 is the point where health outcomes are measured. At baseline, the populations selected are those composed of individuals free from the health outcome of interest, therefore, we consider three different baseline cohorts of individuals. In the case of cancer, the baseline population comprises all individuals who never reported cancer by 2020. In the case of SAH, the baseline population is formed by all individuals who reported, in 2020, enjoying very good or excellent SAH before the pandemic. Hence, we analyse the individuals’ transitions from a general state of good health (defined by the two upper categories of SAH) to a general state of bad health (defined by the two lowest categories of SAH) and associate these transitions with exposure to SUN. Finally, in the case of mortality, the baseline population encompasses all individuals alive at the baseline.

Supported by the prospective cohort design, we rely on two main statistical approaches to identify the association between SUN and the three health outcomes. Firstly, we estimate the relative risk (RR), which reflects the probability of the health outcome among those exposed to SUN relative to the probability of the health outcome among those not exposed to SUN (Merrill 2015). The RR may be biased due to the presence of confounders, therefore, to reduce the impact of these confounders, we regress the health outcomes observed at the end of the study, in 2021 (SC2), on SUN and on a series of control covariates observed at baseline, in 2020 (SC1). Given the binary nature of all health outcomes, we adopt a logistic specification. Ko (2016) also dealt with the issue of simultaneity between health outcomes and SUN by regressing the health outcomes observed at the end of the observation period on covariates observed at the baseline. The set of control variables included in all models is presented above in Table 1. To investigate the possibility of the individual’s socio-economic status influencing the magnitude of the association between SUN and health outcomes, we include in the list of covariates the interaction between SUN (Global) and income and between SUN (Global) and education level.

In all logit specifications, the measure of association between SUN and the health outcome that is estimated, and reported, is the odds ratio (OR). The OR of a given exposure factor is the ratio between the odds of the (health) outcome taking place given that the factor is present and the odds of the (health) outcome occurring given that the factor is absent. Define *P*(*y* = 1/*x*) as the probability of the (health) outcome occurring, conditional on covariates ***x***. In a logistic model, the probability of the outcome is given by $P_{1} = P\left( {y = 1{|}x} \right) = \frac{{e^{{x^{\prime}\beta }} }}{{1 + e^{{x^{\prime}\beta }} }}$, and $P_{0} = P\left( {y = 0{|}x} \right) = \frac{1}{{1 + e^{x^{\prime}\beta } }}$. The odds are then defined as $\frac{{P_{1} }}{{P_{0} }} = e^{x^{\prime}\beta }$, where ***x′β*** is a linear function of the covariates (***x***) and parameters (***β***). It is straightforward to conclude that for a specific exposure factor *E,* whose coefficient from the logistic regression is, say, *β*_2_, the corresponding OR is given by $OR_{E} = e ^{{\beta_{2} }}$. However, when ***x′b*** also includes interaction terms, the interpretation deserves special caution. Assume that $x^{\prime}b = \beta_{0} + \beta_{1} x_{1} + \beta_{2} E + \beta_{12} x_{1} *E + \beta_{3} d + \beta_{13} dE + \beta_{k} x_{k}$, where *x*_*1*_ and *d*, are respectively, a continuous and a dummy variable, *E* is the exposure variable (assumed binary) and ***x***_*k*_ is a list of control variables. Then the OR of exposure *E* is given by $OR_{E} = e^{{\beta_{2} + \beta_{12} x_{1} + \beta_{13} d}}$, thus depending on the covariates *x*_1_ and *d*, in addition to the parameters. To estimate the overall OR_*E*_ we compute the average response of all individuals. The interaction effect of the continuous variable *x*_1_, that is, the effect of the continuous covariate *x*_1_ on OR_*E*_, is given by the derivative of the OR_*E*_ in relation to *x*_1_ $\frac{d}{{dx_{1} }}\left( {e^{{\beta_{2} + \beta_{12} x_{1} + \beta_{13} d}} } \right) = \left( {e^{{\beta_{2} + \beta_{12} x_{1} + \beta_{13} d}} } \right){*}\beta_{12}$. Again, the interaction effect is calculated as the average response of all individuals. To assess the effect of the dummy variable *d* on the OR_*E*_, we calculate the average effect of ${\text{OR}}_{E}$(*d* = 1)–${\text{OR}}_{E}$(*d* = 0). In summary, we run six logistic regression models. The first three models regress the three health outcome measures (cancer, mortality and SAH Poor|Fair_Pand) on SUN, including, simultaneously, as main covariates the different motives of unmet healthcare needs, along with the control variables presented in Table 1. The second group of models regress the three health outcome measures on SUN (Global), but now including the interaction between income, and education, with SUN Global, in addition to all control variables listed in Table 1. Any dataset case that had a missing value in one variable of interest was dropped from the analysis. We used Stata 16.1 for data processing and statistical analysis. To compute the average OR effects, we used the *margins* instruction available in Stata. Stata do-files are available upon request.

## Results

Table 2 displays the prevalence of SUN in both waves, 2020 and 2021. Regarding SUN (Global), there is a clear decrease from 2020 to 2021 of about 12 percentage points (p.p.). Disaggregating by motive, it is noticeable that in both years SUN caused by cancellations/postponements are the most prevalent, followed by fear of getting infected. For the former, the prevalence of SUN falls to less than half in 2021, whereas for SUN due to fear of infection the prevalence in 2021 is about 75% of that in 2020. The prevalence of SUN due to unavailable care is by far the lowest and the more stable in both waves.

**Table 2:** Prevalence of subjective unmet healthcare needs (SUN) in 2020 and 2021

Type of SUN	2020 (%)	2021 (%)
SUN (Fear)	11.64	8.93
SUN (Postponement)	24.96	11.94
SUN (Unavailable care)	5.40	5.36
SUN (Global)	33.41	21.62
*N*	57,041	48,868

Prevalence rates are based on the full sample observed in SC1 (57,559) and in SC2 (49,253). There were missing observations on SUN variables and on the weights; excluding them led to a sample size of 57,041 and 48,868 observations for 2020 and 2021, respectively. The displayed prevalence rates are weighted

Table 3 reports the incidence of SUN in 2021, for two distinct groups: those who reported SUN in 2020 and those who did not.

**Table 3:** Incidence of subjective unmet healthcare needs (SUN) in 2021

	SUN = YES 2020 (%)	SUN = NO 2020 (%)	*N* (Yes; No)
SUN (Fear)	26.8	6.5	5900; 42,029
SUN (Postponement)	26.8	7.1	12,875; 35,054
SUN (Unavailable care)	20.3	4.5	2508; 45,421
SUN (Global)	38.1	13.5	16,985; 30,944

Estimates are based on the full sample of 48,356 respondents who participated in both SC1 and SC2. There were some missing values on SUN variables, for SC1 and SC2, and on weights for SC2, that had to be excluded, thus leading to a sample size of 47,929 observations. The figures presented are weighted (based on weights from SC2)

Considering the new cases of SUN in 2021, the share of such cases related to previous reporting of SUN is between 4 and 4.5 times higher (depending on the motive) when compared to absence of SUN in 2020.

Table 4 displays the prevalence and incidence of the three health outcomes observed in 2021.

**Table 4:** Prevalence and incidence of health outcome variables in 2021

	Prevalence (%)	Incidence (%)
Cancer *N*	5.07 48,046	1.94 42,509
SAH Poor|Fair_Pand *N*	36.03 48,150	9.46 11,234
Mortality *N*	–	2.41 49,555

The figures presented are weighted (based on weights from SC2) and are based on individuals who had valid (non-missing) outcome measures. The “Prevalence” column is based on the full sample and the “Incidence” is based on the baseline populations defined above

The estimates are based on the 48,356 SHARE respondents who participated in both SC1 and SC2. There were some missing values on the outcome measures and on weights, for SC2, and excluding these observations led to sample sizes of 48,046 and 48,150 observations, respectively, for the outcomes Cancer and SAH—these samples were used to create the column “Prevalence” of Table 4. The figures for “Incidence” are based on the full baseline samples (Cancer: 43,693; SAH: 11,396 and Mortality: 49,555 observations). Due to missing values either on one outcome measure or on the weights, at SC2, the final sample is reduced to 42,509 in the case of the cancer baseline and to 11,234 in the case of the SAH baseline. The sample to estimate Mortality comprises all respondents who participated in both SC1 and SC2—48,356, plus those who participated in SC1 and died before the second wave—1199 individuals—amounting to 49,555 observations. The displayed figures are weighted (based on weights from SC2).

As shown in Table 4, the prevalence of cancer in 2021 was 5.07%; however, the incidence was 1.94%. The incidence of cancer in the COVID-19 era is in line with the incidence observed in previous waves of SHARE (own estimates based on all waves of SHARE, available upon request). The prevalence of poor or fair health is 36%, though the new cases for those conditions, in 2021, are 9.46%. The mortality rate was estimated at 2.41%.

Table 5 displays the RR of the health outcomes in 2021 both for the individuals who were and were not exposed to unmet needs in 2020.

**Table 5:** Unadjusted Relative Risk

	Cancer	Mortality	SAH Poor|Fair_Pand
SUN (Fear)	**1.30** [1.08–1.57]	**0.68** [0.562–0.838]	**1.30** [1.10–1.54]
SUN (Postponement)	**1.32** [1.15–1.51]	**0.77** [0.671–0.881]	**1.16** [1.01–1.32]
SUN (Unavailable care)	**1.61** [1.28–2.05]	0.95 [0.729–1.224]	1.30 [0.99–1.69]
SUN (Global)	**1.32** [1.16–1.51]	**0.69** [0.607–0.782]	**1.23** [1.09–1.38]
*N*	42,609	49,466	11,308

95% confidence intervals in brackets. In bold, RR statistically significant at 5%

All estimates are based on the full sample for each baseline population (Cancer: 43,693; Mortality: 49,555, and SAH: 11,396 observations). All cases with missing values on any of the unmet measures were dropped, leading to the final sample sizes presented in this table

As shown in Table 5, the RR for cancer is higher for the exposed to unmet needs (RR greater than one and statistically significant for all motives of SUN). Poor or fair SAH is also higher among those individuals exposed to SUN in 2020 and the difference is statistically significant, except for the case of SUN due to unavailability of care. Differently, in the case of mortality, the RR is higher among the non-exposed to SUN in 2020 (not significant for SUN due to unavailability of care). We estimated the RR by gender and by age categories and did not find any statistically significant differences.

These univariate estimates suggest that exposure to SUN is associated with all health outcomes considered; however, these results might be biased and influenced by the presence of confounding variables, therefore, we next present the results from the estimation of the multivariate model, controlling for some important independent variables.

Firstly, to check if the baseline populations substantially differ from the whole population, Table 6 presents some descriptive statistics that assist in the characterisation of both.

**Table 6:** Summary statistics of the baseline populations, according to SUN (Global) exposure in 2020

	Whole population			Cancer^a^		SAH Poor|Fair_Pand^b^	
	All	Exposed	Not exposed	Exposed	Not exposed	Exposed	Not exposed
Male (%)	41.7	38.0	43.8	37.5	43.3	38.4	45.1
Age	70.4	70.5	70.4	70.0	69.9	67.6	66.9
Educ low (%)	33.5	31.7	34.5	31.7	34.3	18.2	21.0
Educ med (%)	43.3	42.4	43.8	42.4	44.1	42.2	46.4
Income (€)	1342.7	1375.7	1324.4	1365.9	1333.5	2016.6	1762.5
Lives alone (%)	24.8	26.0	24.2	25.7	23.8	22.9	19.9
Big city (%)	16.6	18.0	15.8	17.8	15.9	17.1	17.6
Suburbs (%)	8.7	9.8	8.1	9.5	8.1	13.4	10.7
Large town (%)	16.0	16.5	15.6	16.5	15.7	15.5	16.3
Small town (%)	23.2	22.7	23.5	22.4	23.4	23.1	23.0
SAH Poor_Pre_Pand (%)	7.0	8.2	6.3	6.9	5.1	–	–
SAH Fair_Pre_Pand (%)	26.6	30.5	24.5	29.3	23.4	–	–
SAH Good_Pre_Pand (%)	43.6	42.1	44.5	43.4	45.4	–	–
SAH Very Good_Pre_Pand (%)	16.1	14.0	17.3	14.7	18.3	72.7	69.7
n_Chronic	2.72	3.17	2.46	2.98	2.30	2.03	1.51
Diabetes (%)	18.2	21.5	16.3	21.0	15.6	10.3	8.0
*N*	37,409	13,357	24,052	11,261	21,340	2558	5907

These summary statistics were computed based on the samples used to estimate the regression models

^a^The baseline population for cancer are individuals who never reported cancer until 2020

^b^The baseline population for SAH Poor|Fair_Pand are individuals with SAH very good or excellent before the Corona outbreak

There are no marked differences between the exposed and the non-exposed, within each baseline population. Because SHARE targets older individuals, it is understandable the absence of great discrepancies. The percentage of individuals reporting fair or poor SAH, before the pandemic, is about 8 p.p. higher among the exposed to SUN (Global) than among the non-exposed, both in whole population and among individuals without cancer up to 2020. The average number of chronic conditions and percentage of individuals with diabetes is also higher among the exposed, for all baseline populations.

As shown in Table 7 (models 1 to 3), for cancer and poor or fair SAH, there is a positive association between SUN and worse health outcomes, though OR are not statistically significant. In model 2, there is still evidence of a negative association between SUN and mortality, statistically significant for unmet needs due to postponement of care. Individuals who had healthcare postponed in 2020 were 22% less likely to have died by 2021. Regarding the effects of other covariates on outcomes, males are 1.5 and 2.8 times more likely to have reported cancer and died in 2021, respectively. Age is positively associated with all health outcomes. Low education, compared with high education, is positively associated with mortality and poor/fair SAH. In the latter case, the effect is quite pronounced, and even the effect of medium education makes a difference, compared with high education. The associations between poor or fair SAH and both cancer and mortality are statistically significant. In model 2, the magnitude of this association is striking. In model 3, individuals with very good SAH in 2020 were 25% more likely to report poor/fair SAH in 2021 than individuals with excellent health in 2020. Odds ratio for income are slightly lower than one for all outcomes but are not statistically significant, while the area of residence and living alone show mixed signals (also not significant).

**Table 7:** Association between unmet healthcare needs—SUN (Fear), SUN (Postponement), and SUN (Unavailable care)—and health outcomes—cancer, mortality and poor or fair SAH

	Model 1: cancer		Model 2: mortality		Model 3: SAH Poor|Fair_Pand	
	OR	CI (95%)	OR	CI (95%)	OR	CI (95%)
SUN (Fear)	1.16	[0.934–1.441]	0.782	[0.609–1.004]	1.235	[0.976–1.564]
SUN (Postponement)	1.163	[0.979–1.382]	**0.78**	[0.652–0.934]	0.976	[0.813–1.171]
SUN (Unavailable care)	1.291	[0.963–1.732]	0.948	[0.684–1.313]	1.18	[0.812–1.715]
Male	**1.524**	[1.307–1.777]	**2.817**	[2.408–3.295]	1.023	[0.876–1.194]
Age	**1.022**	[1.012–1.031]	**1.097**	[1.087–1.107]	**1.026**	[1.016–1.037]
Educ_low^a^	0.919	[0.737–1.147]	**1.309**	[1.030–1.663]	**2.054**	[1.641–2.570]
Educ_med^a^	0.912	[0.750–1.110]	1.181	[0.940–1.483]	**1.363**	[1.122–1.656]
Income	0.995	[0.952–1.041]	0.916	[0.802–1.046]	0.918	[0.841–1.002]
Lives alone	0.942	[0.785–1.130]	0.992	[0.836–1.177]	1.111	[0.926–1.334]
Big city^b^	1.014	[0.808–1.273]	0.832	[0.655–1.057]	0.913	[0.725–1.149]
Suburbs^b^	0.757	[0.550–1.042]	0.963	[0.712–1.301]	0.757	[0.576–0.995]
Large town^b^	0.908	[0.718–1.149]	1.011	[0.811–1.261]	0.844	[0.668–1.067]
Small town^b^	0.984	[0.803–1.205]	1.003	[0.831–1.211]	0.818	[0.665–1.007]
SAH Poor_Pre_Pand^c^	**2.48**	[1.604–3.837]	**9.275**	[5.159–16.67]	–	–
SAH Fair_Pre_Pand^c^	**1.534**	[1.052–2.237]	**3.044**	[1.715–5.402]	–	–
SAH Good_Pre_Pand^c^	1.213	[0.851–1.729]	1.466	[0.828–2.596]	–	–
SAH Very Good_Pre_Pand^c^	1.182	[0.811–1.723]	1.132	[0.600–2.135]	**1.258**	[1.056–1.500]
n_Chronic	1.02	[0.972–1.070]	1.037	[0.997–1.078]	**1.422**	[1.347–1.501]
Diabetes	1.028	[0.839–1.259]	**1.211**	[1.023–1.435]	1.192	[0.952–1.494]
N	32,601		37,409		8465	
LogL	− 3373.34		− 3312.06		− 2475.33	
Pseudo R2	0.0285		0.1977		0.1086	

All models include dummies for countries

In bold, OR statistically significant at 5%

*Reference categories*

^a^Educ_high

^b^Rural

^c^SAH Excellent_Pre_Pand

Models 4 to 6, shown in Table 8, include, in addition to the covariates from models 1 to 3, the interaction terms between income (or education) and SUN (Global). The main noticeable change is that the association between SUN and cancer becomes statistically significant. Individuals who experienced any kind of SUN in 2020 were 27% more likely to report cancer in 2021. For mortality, there is still a negative (slightly reinforced) association between SUN and this health outcome. For SAH, the OR is greater than one but again not significant. For cancer and SAH, the interaction terms show in general the expected signs (higher education and higher income weaken the association between SUN and worse health) but the coefficients are not statistically significant. Again, for mortality, results are the opposite. If anything, individuals with higher education or higher income, reporting SUN in 2020, were less likely to have died by 2021, compared with individuals with lower education/income, also reporting SUN in 2020. Still, coefficients are not statistically significant. We do not report OR for the remainder covariates as there are no differences worth mentioning, compared with results in Table 7.

**Table 8:** Association between SUN (Global) vs health outcomes and interaction effects

	Model 4: cancer	Model 5: mortality	Model 6: SAH Poor|Fair_Pand
*Odds ratio*			
SUN (Global)	**1.27** [1.068;1.472]	**0.743** [0.601;0.885]	1.081 [0.897;1.265]
*Marginal effects of interaction terms*			
Educ_low × SUN (Global)	− 0.025 [− 0.456;0.506]	0.001 [− 0.329;0.329]	− 0.374 [− 0.843;0.094]
Educ_med × SUN (Global)	0.262 [− 0.220;0.744]	0.092 [− 0.250;0.435]	− 0.109 [− 0.575;0.356]
Income × SUN (Global)	− 0.045 [− 0.194;0.103]	0.024 [− 0.112;0.161]	− 0.139 [− 0.296;0.170]

The coefficients are adjusted for Age, Male, Educ_low, Educ_med, Income, Lives alone, Big city, Suburbs, Large town, Small town, SAH, n_Chronic, Diabetes and country dummies

95% Confidence intervals in brackets. In bold, statistically significant coefficients at 5%

## Discussion

The COVID-19 pandemic generated unprecedented levels of SUN (Arnault et al. 2021). Our results show a pronounced decrease in SUN, due to cancellation/postponement and fear of infection, from 2020 to 2021. Multiple causes can account for this decrease, including the relaxation of some lockdown measures, on the one hand, that allowed an improved healthcare service response and, on the other, the positive evolution of the vaccination rates, which allowed individuals to progressively seek medical care without the fear of being infected by the coronavirus. Notwithstanding this decline in SUN rates, the figures obtained for 2021 are well above average values obtained from prior waves of SHARE (own calculations), 12.3% and 9.7%, in 2015 and 2017, respectively. But we are mostly interested in associations between SUN in 2020 and health outcomes observed in 2021.

The results show that reporting SUN in 2021 is more likely among individuals who reported SUN in 2020 as well, compared to individuals with no SUN in 2020, suggesting some persistence of SUN over time. In the pandemic context, a large proportion of unmet healthcare needs come from cancellations or postponement of planned healthcare. Thus, many individuals with SUN in 2020 went without previously scheduled care and therefore are expected to have medical appointments, or seek medical attention, again in 2021. For this reason, they are more exposed to SUN in 2021. Differently, those who did not report SUN in 2020 either did not seek healthcare/did not have any appointment or had their healthcare needs met. This might mean that they are healthier, not needing healthcare also in 2021, or that they are sicker individuals, and for this reason they were prioritised in the pandemic context, being less likely to see their appointments cancelled or demands denied both in 2020 and in 2021.

Regarding the association of SUN in 2020 with health outcomes in 2021, the statistical analysis suggests a positive association with poor/fair SAH and cancer, and a negative association with mortality. Controlling for possible confounders, the multivariate analysis shows that these results hold for mortality, but the association loses statistical significance regarding SAH. In the case of cancer, the positive association with SUN only appears in the model with SUN (Global) (model 4). The association between SUN and cancer is in accordance with the few studies on the positive association between SUN and worse health (Ko 2016; Ju et al. 2017; Gibson et al. 2019). Our results suggest that new diagnoses of cancer in 2021 are more likely among individuals who missed scheduled appointments than among individuals who either did not have/did not seek any appointment or did not forego planned healthcare, in 2020. Some screening modalities have the potential to detect and remove cancer precursors, such as those for colorectal and cervical cancers. In these situations, screening itself can reduce cancer incidence (Lauby-Secretan et al. 2018). Still, for most cancer cases, it is unlikely that the absence of cancellation/postponement of medical appointments and treatments in 2020 would have prevented the new cases of cancer in 2021. However, there might have been a delay in diagnosis, and this can have serious health consequences. Depending on cancer type and location, delaying cancer screening and preventive services by six weeks is problematic; delaying by six months may lead to dramatic increases in cancer death rates (Meyer et al. 2020; Sud et al. 2020). When it comes to SUN, there is always the discussion on whether unmet needs are true needs. Our results suggest that these individuals really needed healthcare.

Concerning the association between SUN and mortality, our results differ from previous evidence (Zhen et al. 2015; Lindström et al. 2020). This might be explained by the specificity of unmet healthcare needs during the pandemic. Many of these SUN were due to the cancellation or postponement of, and non-attendance to, planned appointments. Because healthcare systems were forced to prioritise patients, our results suggest that patients at greater risk of death were given priority and were less likely to experience SUN, either due to their own decision or as the result of the health system response to the COVID-19 crisis.

While in the case of mortality, the evidence obtained suggests that health systems prioritised life-threatening conditions, in the case of cancer, health systems seem to have failed to address patients’ needs. It is not possible to know the full extent of the consequences of these SUN. It depends on the delays of diagnosis, but cancer-related mortality might increase in the future (Richards et al. 2020; Sud et al. 2020). Based on previous evidence, a stronger association between SUN and health outcomes was expected among individuals with higher levels of education and income. However, the interaction terms obtained in this study are not statistically significant. This result is reassuring from the perspective of health inequalities, which could widen if richer and more educated individuals had greater chances to resume cancer screening (and hence, greater chances of reporting a diagnosis of cancer in 2021) or if they had greater chances of being prioritised during the pandemic (showing therefore a stronger negative association between SUN and mortality).

Some limitations apply to our analysis namely using data from an observational study which can jeopardise the validity of our results as causal estimates of the impact of exposure to SUN on health outcomes. The follow-up period is one year which may not be sufficient to fully capture the association between SUN and health status. Still, there are no certainties on the optimal follow-up time. Zhen et al. (2015) conjecture that the mortality risks associated with unmet needs may be substantially higher if examined over a period longer than three years. On the other hand, Gibson et al. (2019) argue that an annual panel would allow for more flexibility to test a more immediate effect of SUN. Kim et al. (2019) say that the time lag between unmet needs and the manifestation of self-rated poor health may not be as long as a year. The exposure status (SUN) may not fully and properly measure unmet healthcare need. The only information provided by the dataset is whether the individual had an unmet health care need at the baseline, but nothing is known about the length of the exposure to SUN, and if the unattended medical treatments were recovered. Nonetheless, there is evidence that waiting times alone can cause harm to individuals’ health (Moscelli et al. 2016). Our results are valid for individuals who never had cancer and started at baseline with an excellent or very good health. This might explain why, unlike in the previous studies, we did not find an association between SUN and SAH. As noted by Gibson et al. (2019), citing Heckman (1981), if deterioration of health at time *t* (within the period analysed) is affected by baseline health status, prior to the beginning of the follow-up period (that is, if health deteriorates slower, *ceteris paribus*, among those with better initial health), we might have obtained a biased estimate of the true effect of SUN on the deterioration of health, measured by SAH. Ko (2016), for example, did not find evidence of a significant association of unmet needs due to mild symptoms with health outcomes. Although the available data does not allow us to distinguish mild symptoms from the remainder, it is likely that SUN among individuals with excellent or very good SAH concerned not so severe situations, otherwise, the system would have prioritised them. In addition, the baseline population for the SAH analysis is restricted to 8465 individuals which represents a drop of about 77% of the sample. It is also possible that among older individuals the association between SUN and health is more visible for specific symptoms, such as fatigue, falling or dizziness. Bergeot and Jusot (2022) analysed these conditions, obtaining clearer results than ours regarding the association between unmet health care needs and deterioration of health. Also, in future research, other models (e.g. fixed effects) might be used to explore the association between SUN and SAH, mortality and cancer.

Despite the limitations, the study population is large, and our work not only contributes to an under researched topic, but also, to the best of our knowledge, it is also the first to analyse the association between SUN and cancer prevalence. It further contributes to a better understanding of the consequences of the pandemic. Several studies relied on SC1 to identify the predictors of SUN during the first wave of the pandemic, but our study goes a step forward investigating what health implications might follow from the unprecedented levels of SUN during the first months of the pandemic. During public health crises, like the COVID-19, it is important, firstly to ensure that individuals do not shy away from health services due to fear, avoiding unmet needs for this reason. And, secondly, health systems should not lose sight of patients affected by conditions other than the disease responsible for wide outbreaks. Perhaps, the lasting effect of what we learned now will depend on how far we are from the next worldwide pandemic.

## Appendix

**Table:**

Variable	Source of data
*Health outcome measures*	
Cancer	All waves of SHARE: the baseline population, in 2020, consists of individuals who, in the regular waves of SHARE, never selected the option ‘Doctor told you had: cancer’, and, in SC1 wave, did not select the option “Cancer or malignant tumor, including leukemia or lymphoma” in the question “Since we last interviewed you, were you diagnosed with a major illness or health condition?”. We used the SC2 wave to identify individuals with cancer in 2021
Mortality	xt module released jointly with SC2 data (sharewX_rel8-0-0_gv_allwaves_cv_r file)
SAH	For the baseline population, in 2020, and controls, data come SC1. For the outcome, in 2021, data come from SC2
*SUN as measure of exposure*	
SUN (all motives)	To identify exposure to SUN we used data from SC1; data from SC2 were used simply to compute some descriptive statistics
*Control variables*	
Age	SC1
Male	SC1
Education	Data come from the last regular wave of SHARE in which the individual was interviewed and had level of education measured
Income	Information about household income comes from SC1, obtained from the question “How much was the overall monthly income, after taxes and contributions, that your entire household had in a typical month before Corona broke out?”. To compute the equivalent income, information about the household size was extracted from the file CV_R file released along with the SC1 wave
Lives alone	SC1—module CV_R
Place of residence	Data come from the last regular wave of SHARE in which the individual was interviewed and had place of residence registered
n_Chronic	The information about chronic diseases before the Corona outbreak comes from regular waves of SHARE—gv_health module. This number adds up with newly diagnosed chronic diseases reported in SC1
Diabetes	All regular waves of SHARE and SC1, depending on when the disease was diagnosed

*SC1* SHARE Corona Survey 2020, *SC2* SHARE Corona Survey 2021.
